# Municipal solid waste landfill leachate treatment by fenton, photo-fenton and fenton-like processes: Effect of some variables

**DOI:** 10.1186/1735-2746-9-3

**Published:** 2012-08-02

**Authors:** Mohammad Ali Zazouli, Zabihollah Yousefi, Akbar Eslami, Maryam Bagheri Ardebilian

**Affiliations:** 1 Department of Environmental Health Engineering, Faculty of Health and Health Sciences Research Center, Mazandaran University of Medical Sciences, Sari, Iran; 2 Department of Environmental Health Engineering, School of Public Health, Shahid Beheshti University of Medical Sciences, Tehran, Iran

**Keywords:** Advanced oxidation processes, Photo-Fenton, Fenton, Fenton-like, Landfill leachate

## Abstract

Advanced oxidation processes like Fenton and photo-Fenton have been effectively applied to oxidize the persistent organic compounds in solid waste leachate and convert them to unharmful materials and products. However, there are limited data about application of Fenton-like process in leachate treatment. Therefore, this study was designed with the objective of treating municipal landfill leachate by Fenton, Fenton-like and photo–Fenton processes to determine the effect of different variables, by setting up a pilot system. The used leachate was collected from a municipal unsanitary landfill in Qaem-Shahr in the north of Iran. Fenton and Fenton-like processes were conducted by Jar-test method. Photo-Fenton process was performed in a glass photo-reactor. In all processes, H_2_O_2_ was used as the oxidant. FeSO_4_.7H_2_O and FeCl_3_.6H_2_O were used as reagents. All parameters were measured based on standard methods. The results showed that the optimum concentration of H_2_O_2_ was equal to 5 g/L for the Fenton-like process and 3 g/L for the Fenton and photo-Fenton processes. The optimum ratio of H_2_O_2_: Fe^+2^/Fe^+3^ were equal to 8:1 in all processes. At optimum conditions, the amount of COD removal was 69.6%, 65.9% and 83.2% in Fenton, Fenton-like and photo–Fenton processes, respectively. In addition, optimum pH were 3, 5 and 3 and the optimum contact time were 150, 90 and 120 minutes, for Fenton, Fenton-like and photo–Fenton processes, respectively. After all processes, the biodegradability (BOD_5_/COD ratio) of the treated leachate was increased compared to that of the raw leachate and the highest increase in BOD_5_/COD ratio was observed in the photo-Fenton process. The efficiency of the Fenton-like process was overally less than Fenton and photo-Fenton processes, meanwhile the Fenton-like process was at higher pH and did not show problems.

## Introduction

Currently, deposition of municipal and industrial wastes in landfills is considered as the most widespread and economical method for waste disposal. Besides, the scarcity of the available landfill sites and the large amount of generated leachate from these sites are the major problems of municipal solid waste disposal with landfilling method [1]. The municipal solid waste (MSW) gene-ration was more than ten million tons per year in Iran that about 84% of generated MSW was disposed in the landfill [2]. One of the main problems of solid waste landfilling is leachate production. Leachate contains various pollutants such as high concentrations of organic matters (biodegradable or non-biodegradable) and inorganic constituents. The landfill leachate discharge may lead to serious environmental problems. Leachate may percolate through soils and subsoils, causing extensive pollution in surface and ground water resources if they are not properly treated and safely disposed. Thus, it should be treated before discharge in the environment or to a sewage system for treatment [3-5].

The selection of the type of treatment process will depend largely on the contaminants to be removed because of the high variable quantity and quality of landfill leachates [3,5]. Precipitation process (coagulation, flocculation, and sedimentation) is the most common method of removing soluble metals and many anionic species [5]. The main task of leachate biological process is reducing the organic biodegradable compounds. Biological processes can be effective for young leachates with high BOD_5_/COD ratio [6]. Thus, to remove the readily biodegradable organic pollutants in young leachate, the biological treatments are usually preferred over physico-chemical. However, in the case of "old" leachates, biological process cannot successfully treat the leachate because it contains a higher proportion of the organic matter and consists of relatively refractory compounds or nonbiodegradable matters and these pollutants inhibit biomass activity and/or are recalcitrant to biological treatments. In such instances, leachate must usually be treated by more expensive physico-chemical treatments such as chemical precipitation, adsorption on activated carbon, chemical oxidation and etc., to obtain satisfactory treatment efficiencies. Among them, growing interest has been focused on advanced oxidation processes (AOPs) [1,7].

In general, AOPs are defined as oxidation processes which generate hydroxyl radicals in sufficient quantity [7]. AOPs are chemical, photochemical, photocatalytic and electrochemical methods characterized by in situ generation of hydroxyl radical [8,9]. Hydroxyl radical has a very high oxidation potential and is able to oxidize almost all organic pollutants [10]. These processes are also described by other researchers [11].

Most of the AOPs use a combination of strong oxidants like O_3_ and H_2_O_2_ with catalysts like transition metals, iron, semiconductor solids, and radiation or ultrasound. Typical AOPs include O_3_/UV, H_2_O_2_/UV, TiO_2_/UV, H_2_O_2_/Fe^2+^ and H_2_O_2_/Fe^2+^/UV [12]. Sonolysis is also considered as an advanced oxidation process because of hydroxyl radical generation by pyrolysis of water molecules during cavitations. It was effectively used for removing low concentration of organic pollutants such as trichloroethylene (TCE) from aqueous streams [13].

Fenton process is one of the non-photochemical advanced oxidation processes that starts with aqueous mixtures of Fe (II) and hydrogen peroxide. It has been demonstrated that Fenton’s reagent is able to destroy different phenols, nitrobenzene, herbicides, Di-(2-Ethylhexyl) phthalate (DEHP), and Linear Alkylbenzene Sulfonate (LAS) in water media, as well as reducing COD in municipal wastewater [14-17]. This process generates hydroxyl radicals (OH˚) which are highly oxidative. The hydroxyl radical attacks, degrades, and mineralizes the organic pollutants to carbon dioxide and water [16-18]. Photo-Fenton is a process in which hydroxyl radicals are produced through the decomposition of hydrogen peroxide using Fe(II) or Fe(III) in acidic conditions under UV radiation or visible light [19]. Fenton-like reaction was firstly started with Fe(III) and H_2_O_2_[18].

All these processes, except the Fenton-like process, have been recently reviewed by Renou *et al.,* confirming that AOPs, adapted to old or well-stabilized leachate, may be applied to oxidize organic substances to their highest stable oxidation states, carbon dioxide and water, and to improve the biodegradability of recalcitrant organic pollutants up to a value compatible with subsequent economical biological treatment [20]. The applicability of photo-Fenton and Fenton processes for landfill leachate treatment have previously been reported in literatures [7,11,12,14,18,20-24]. The study about the biodegradability enhancement of landfill leachate by Fenton oxidation showed that this process improved the biodegradability of leachate [25]. Other researchers performed various studies about landfill leachate treatment by Fenton process and due to the characteristic of lea-chate and conditions of each study, different results have been obtained [7,18,22-24,26]. Although several studies have been done on the treatment of landfill leachates by Fenton and photo-Fenton processes, there has been little effort to optimize the operation conditions to achieve the maximum removal efficiency. Moreover, there have been few studies carried on photo-Fenton process in leachate treatment. Furthermore, there are no data about the applicability of the Fenton-like process for leachate treatment. Therefore, the main objectives of the present work were to investigate the old landfill leachate treatment by Fenton, Fenton-like, and photo-Fenton processes in order to determine the effect of different parameters on treatment efficiency and to optimize the operation conditions.

## Materials and methods

### Leachate sampling

Leachate samples were collected from an old municipal landfill located in Qaem-Shahr, a city in the north of Iran. It is located near Tellar River and receives approximately 250 tons of municipal solid wastes per day. At the lowest point of the landfill, leachate exits from the surface forming an artificial pond. In this study, raw leachate samples were collected from the artificial pond in 20 L sealed plastic barrels, transported to the laboratory and stored at refrigerator before being used and analyzed.

### Pilot system and used apparatus

**Figure 1:**
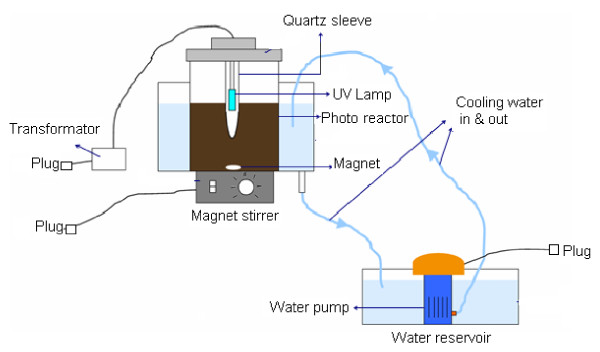
Scheme of used pilot for photo-Fenton process.

The Fenton and Fenton-like processes were conducted by Jar-test device model M.K. 2001 at 100 rpm [27] at various times. The photo-Fenton process was performed in 2-liter Pyrex glass photo-reactor (Figure 1). Agitation in the reactor was done with magnet and magnet stirrer [12]. A 150-watt medium-pressure UV lamp was placed in a quartz sleeve and was immersed in the middle of the reactor. Lamp intensity was 1.5-1.7 w/m^2^, determined by Hagner radiometer model EC1-X. The lamp intensity was measured at different times from a distance of 7 cm. The peak wavelength of UV lamp was 247.3 nm and its type was UV-C. Aluminum foil was used to cover around the reactor to protect the laboratory staff against UV radiation. The reactor was surrounded by a water cooling jacket to maintain constant temperature [11,12]. The experiments were conducted at room temperature controlled by a water bath, and a water pump was used for pumping water. 

### Experimental procedures and analytical methods

The sample plastic barrels were thoroughly shaken for re-suspension of possibly settled solids and 1000 mL of leachate was transferred to the beakers or reactor. Then, the pH of the solution was adjusted by adding either NaOH or H_2_SO_4_ solutions. The desired concentration of Fe(II) (in the Fenton and photo-Fenton processes), Fe(III) (in the Fenton-like process) and H_2_O_2_ were added to the samples, respectively. After each process and settling period (1 hour), the supernatant was used for chemical analysis which consisted of COD, BOD_5_, Total Kjeldahl Nitrogen (TKN), ortho-phosphorous, Fe, Cu, Pb, Cd and total Cr. All analyses were performed according to Standard Methods for the Examination of Water and Wastewater [28]. To report the COD concentration of the treated samples, interferences caused by residual H_2_O_2_ were avoided [12,29]. To determine the positive intervention of hydrogen peroxide on COD, the control experiments were conducted with similar conditions. The results of these experiments showed that the average increase of COD was 8.5 ± 5.8 percent and the maximum increase was 14.3%.

### Reagents

The materials used in the experiments were FeSO_4_.7H_2_O (in Fenton and photo-Fenton processes) and FeCl_3_.6H_2_O (in Fenton-like process), as catalysts. In all processes H_2_O_2_ 30% was used as oxidant. To adjust pH before any process, H_2_SO_4_ 96% and NaOH 10 M were used [12,30]. All chemicals were selected according to Standard Methods [28] and purchased from Merck Co, Germany.

### Statistical analysis

One-way ANOVA was used (SPSS software) to determine the significant level of differences among the experimental runs. Post hoc multi comparisons were performed using LSD test. When p_value_ was less than 0.05, it was considered that there are significant diffe-rences between the variables.

## Results

### Leachate characteristics

The composition and physicochemical characteristics of the leachate are summarized in Table 1. It was observed that this leachate presents a low value of COD and BOD_5_. In addition, the heavy metals concentration and the BOD5/COD ratio were low.

**Table 1:** Leachate characteristics obtained from the municipal landfill of Qaem-Shahr city

Parameter*	**Unit**	**Value**		
		**Minimum**	**Maximum**	**Mean ± SD**
pH	-	8.24	8.26	8.25 ± 0.01
Temperature	°C	19.50	21.50	20.50 ± 1
COD	mg /L	3923	3995	3960 ± 36
BOD_5_	mg /L	46.50	48.20	47.40 ± 0.85
TKN	mg N/L	502.0	550	532 ± 26
Ortho-phosphorous	mg P/L	91.60	91.65	91.62 ± 0.03
Fe	mg /L	81.0	81.90	81.90 ± 0.5
Cu	mg /L	1.95	2.10	2 ± 0.08
Pb	mg /L	ND	ND	ND
Cd	mg /L	ND	ND	ND
Total Cr	mg /L	2.70	2.78	2.75 ± 0.04

* For all parameters, three samples were analyzed.

ND: no detectable.

### Effect of H_2_O_2_ concentration

**Figure 2:**
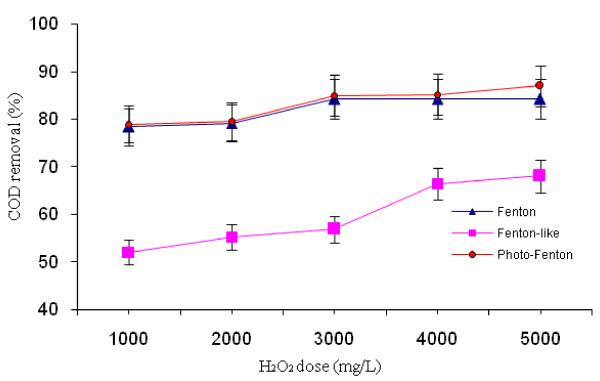
** The effect of H**_**2**_**O**_**2**_**concentration on COD removal by different AOPs.** (Fe^2+^ and Fe^3+^ = 500 mg/L; oxidation time = 60 min; pH = 4).

In order to investigate the optimum H_2_O_2_ concentration, different initial concentrations were tested. The theoretical mass ratio of removable COD to that of H_2_O_2_ is 470.6/1000; that is 1000 mg/L H_2_O_2_ theoreti-cally removes 470.6 mg/L COD by oxidation [18]. Thus, the H_2_O_2_ concentrations from 1000 to 5000 mg/L were used. It should be mentioned that pH range for the Fenton process in different studies was 2–6 [18], so the average of this was considered for the starting of the test. Figure 2 shows the effect of initial hydrogen per-oxide concentration on the final COD removal efficiency. It was observed that COD removal efficiencies increased from 52% to 87% when H_2_O_2_ concentration increased up to 5000 mg/L. The optimal concentration of H_2_O_2_ was obtained as 5000 mg/L for the Fenton-like process, while it was 3000 mg/L for Fenton and photo-Fenton processes. Post hoc comparisons using the LSD test revealed that Fenton-like process showed a significantly less efficiency in COD removal in comparison with Fenton and photo-Fenton processes (p <0.05), but no significant differences were observed between the Fenton and photo-Fenton processes (p >0.05). 

### Effect of H_2_O_2_ to Fe^2+^/ Fe^3+^ ratio

**Figure 3:**
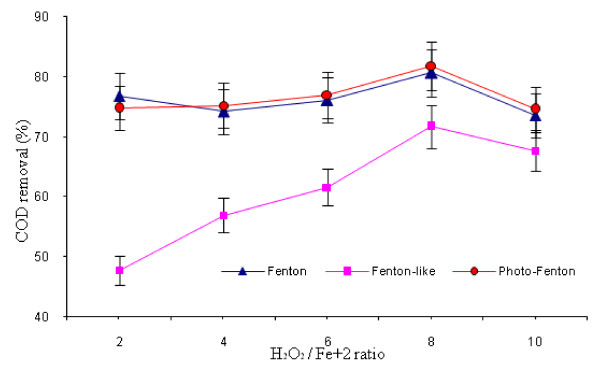
** Effect of H**_**2**_**O**_**2**_** to Fe**^**2+**^**/ Fe**^**3+**^** ratio on COD removal by different AOPs (H**_**2**_**O**_**2**_ **= 3000 mg/L for Fenton and photo-Fenton; H**_**2**_**O**_**2**_ **= 5000 mg/L for Fenton-like; oxidation time = 60 min; pH = 4).**

At this stage, H_2_O_2_ to Fe^2+ +^ ratio in Fenton and photo-Fenton processes and H_2_O_2_ to Fe^3+^ ratio in Fenton-like process were optimized. Several H_2_O_2_ to Fe^2+^/Fe^3+^ ratios equal to 2:1, 4:1, 6:1, 8:1 and 10:1 were tested to investigate the optimum value. These experiments were done with optimal concentration of H_2_O_2_ (5000 mg/L) and pH = 4. The results showed that the optimum ratio of H_2_O_2_ to Fe^2+^ in the Fenton and photo-Fenton processes and H_2_O_2_ to Fe^3+^ ratio in Fenton-like process was 8:1(Figure 3). COD removal was between 71 to 81 percent in the three processes at the optimum rate. The statistical analysis of this effect was similar to the effect of H_2_O_2_ concentration.

### Effect of pH and reaction time

**Figure 4:**
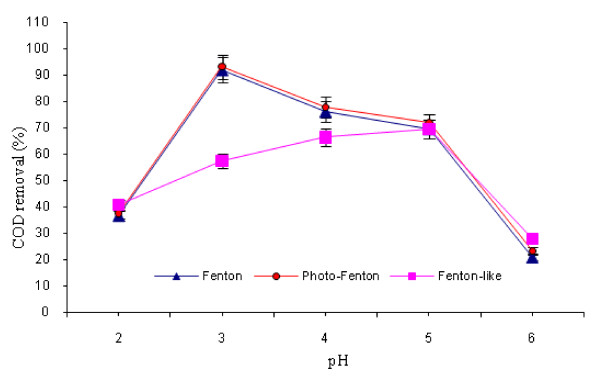
** Effect of pH on COD removal by different AOPs (H**_**2**_**O**_**2**_  **= 3000 mg/L for Fenton and photo-Fenton; H**_**2**_**O**_**2**_ **= 5000 mg/L for Fenton-like; oxidation time = 60 min; H**_**2**_**O**_**2**_**to Fe**^**2+**^**/Fe**^**3+**^**ratio = 8:1)**

**Figure 5:**
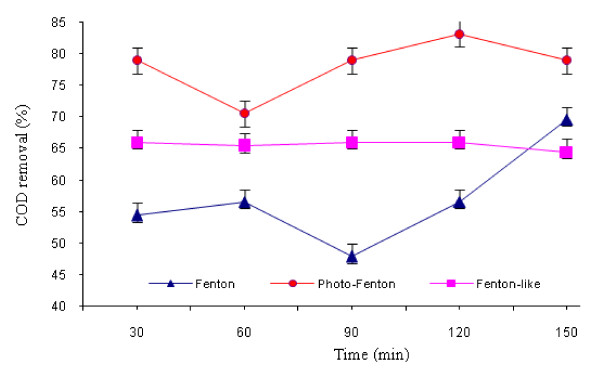
** Effect of oxidation time on COD removal by different AOPs (H**_**2**_**O**_**2**_ **= 3000 mg/L for Fenton and photo-Fenton; H**_**2**_**O**_**2**_ **= 5000 mg/L for Fenton-like; oxidation time = 60 min; H**_**2**_**O**_**2**_**to Fe**^**2+**^**/Fe**^**3+**^**ratio = 8:1 and optimum pH).**

This experiment was conducted at optimum conditions (time = 60 min, H_2_O_2_ to Fe^2+^/Fe^3+^ ratio = 8:1). The results of this experiment are illustrated in Figure 4. Which, it shows that the optimum pH for Fenton, Fenton-like and photo-Fenton processes were 3.0, 5.0, and 3.0, respectively. Figure 5 shows the effect of oxidation time on COD removal in the three processes. As shown in this Figure, the optimum reaction time for Fenton, Fenton-like and photo-Fenton processes were 150, 90 and 120 minutes, respectively. Post hoc comparisons using the LSD test revealed that there were no significant differences between the three treatment methods regarding pH role (p >0.05). However, the effect of reaction time was significantly different between the three methods (p <0.05).

### Biodegradability changes of leachate

**Figure 6:**
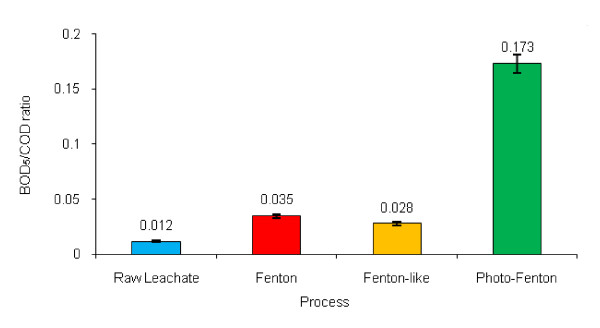
** Variations of BOD**_**5**_**/COD ratio of the treated leachate based on the studied processes.**

The biodegradability of the leachate was evaluated through the evolution of the BOD_5_/COD ratio. The BOD_5_/COD ratio of treated leachate after the studied processes is shown in Figure 6. As shown, the BOD_5_/COD ratio increased in the treated leachate by the three processes. The highest increase was due to the photo-Fenton process.

### Comparison of the treatment processes

Using the previously optimized experimental conditions, the efficiency of the three processes was evaluated toward the removal of COD, TKN, Cu, Cr and ortho-phosphorous variation of iron concentration in the treated leachate (Table 2). In optimum conditions, the removal rate of COD in Fenton, Fenton-like and photo-Fenton processes were 69.6%, 65.9% and 83.2%, respectively. The maximum and minimum removal efficiency of TKN were observed in Fenton and photo-Fenton processes, respectively. In addition, the lowest and the highest rate of phosphate removal was in the Fenton-like and photo-Fenton processes, respectively. In all processes, TKN removal was low in comparison with other parameters, and phosphate removal with over 95% was more than the other parameters.

**Table 2:** COD, TKN, Cu, Cr and ortho-phosphrous removal(%) by the three AOPs in optimum conditions

**Process**	**Removal (%)**				
	**COD**	**TKN**	P*	**Cu**	**Cr**^**#**^
Fenton	69.6	33.2	96.3	35.7	72.7
Fenton-like	65.9	19	95.4	28.6	63.6
Photo-Fenton	83.2	16.8	96.9	50	100

*Ortho-phosphorous.

# total.

## Discussion

### Leachate characteristics

As shown in Table 1, the studied leachate presents a low value of COD and BOD_5_. Taking into account the low concentration of heavy metals, the pH value (8.25) and the low value of the BOD5/COD ratio [(47/3960) = 0.012], the leachate was classified as "old or stabilized or mature" and non-biodegradable leachate [7,10]. The value of the parameters was much higher than the Iranian Standards for discharge (for instance, COD value limit discharge = 100 mg/L) thus, a treatment to remove organic matter was necessary. The low BOD_5_/COD ratio of the leachate indicated that the biological treatment was difficult; therefore, AOPs were studied as alternative treatment options. In most cases, intensive and sophisticated physicochemical processes are necessary for the treatment of aged leachates [10]. In the literature, a considerable variation in the quality of leachate produced from different landfills in the world has been reported. In the previous studies, it has been concluded that leachate from young landfill is characterized by high COD, even several thousands of mg/L, while COD concentrations are below a few hundreds mg/L in leachate from the old landfill [4,5]. The study in Hamadan landfill showed that COD and BOD of the leachate were 75000 and 2500 mg/L, respectively. This indicates that Hamadan landfill leachate was fresh; that is, landfill was young [31]. The municipal landfill of Qaem-Shahr city is an old landfill with more than twenty years old.

### Effect of H_2_O_2_ concentration

As shown in Figure 2, the optimal concentrations of H_2_O_2_ were obtained as 5000 mg/L for the Fenton-like process, but it was 3000 mg/L for the Fenton and Photo-Fenton processes. The COD removal efficiencies increased when H_2_O_2_ concentration increased up to the optimum concentration. H_2_O_2_ concentration was a critical variable in the AOPs. A low concentration of H_2_O_2_ did not generate enough OH^·^ in the solution. Besides, increasing H_2_O_2_ concentration had two opposite effects on the reaction yields [12]. Thus, there was an optimum H_2_O_2_ concentration to achieve the maximum percentage of removal.

### Effect of H_2_O_2_ to Fe^2+^/ Fe^3+^ ratio

As shown in Figure 3, optimum ratio of H_2_O_2_ to Fe^2+^ in the Fenton and photo-Fenton processes and H_2_O_2_ to Fe^3+^ ratio in the Fenton-like process was 8:1. In this study, the rate of COD removal was decreased with increasing the H_2_O_2_ to Fe^2+^/Fe^3+^ ratio more than 8:1. The oxidation process led to an increase in the concentration of hydroxyl radicals (OH^·^) up to a certain concentration, when the hydrogen peroxide ratio increased. The hydroxyl radical has a very high oxidation potential and is able to oxidize almost all organic pollutants. When hydrogen peroxide increased more, it started to react with hydroxyl radicals, acting as a free-radical scavenger itself, decreasing the hydroxyl radicals concentration and generating perhydroxyl radials (HO_2_·) with much less oxidation capacities [12]. Therefore, the removal rate decreased.

### Effect of pH and reaction time

Several authors have demonstrated that pH is one of the major factors limiting the performance of the AOPs in the leachate treatment. The pH solution affects role in controlling the activity of the oxidant and the substrate, the speciation of iron, and the stability of hydrogen peroxide [7,11,18,19,21,24,25]. An essential characteristic of the Fenton process is that pH in the acidic range strongly favors oxidation. Optimal pH values reported for conventional photo- and electro-Fenton processes for landfill leachate treatment range between 2.0 and 4.5 [11]. According to other studies, optimum pH for the Fenton process varies from 2 to 6 [18]. The results of this study showed that the optimum pH for the Fenton, Fenton-like and photo-Fenton processes were 3.0, 5.0, and 3.0, respectively (Figure 4). These results agreed well with those reported by other authors, except that of Hermosilla *et al*; that reported an optimal pH of 2.5 for the treatment of landfill leachate by conventional Fenton and photo-Fenton processes [11].

As shown Figure 5, the optimum reaction time for the Fenton, Fenton-like and photo-Fenton processes were 150, 90 and 120 minutes, respectively. In this case, the Fenton-like process showed more COD reduction than the Fenton process. This was contrary to our expectations. This phenomenon is probably due to the effect of pH, because optimum pH was different in the two processes. However, the COD reduction of the Fenton process exceeded from the Fenton-like process at the end of the experiment. There are no similar data about the effect of reaction time in literature reviews.

### Biodegradability changes of leachate

Initially, the biodegradability of the leachates was evaluated through the evolution of the BOD/COD ratio [10]. The BOD_5_/COD ratio was 0.012 in raw leachate. The BOD_5_/COD ratio of the treated leachate after the studied processes is illustrated in Figure 6. As shown in this figure, the BOD_5_/COD ratio increased in the treated leachate by the three processes. The highest increase was for the photo-Fenton process because photochemical processes can break down and transform the non-biodegradable or refractory organic matter to more biodegradable forms. The other researchers reported similar results and confirmed that photochemical processes can transform the refractory organic matter into easily biodegradable products, improving the efficiency and reducing the cost of further biological steps [10]. Lau *et al* applied the photo-Fenton treatment to increase the BOD_5_/COD of leachate from 0.08 to 0.14 [26]. In the present study, BOD_5_/COD ratio in the Fenton-like process increased from 0.012 to 0.028. Those results were consistent with the findings of other studies, for example, Kim *et al* have reported that leachate BOD_5_/COD raised from 0.14 to 0.27 by Fenton-like process [21]. However, little research has been conducted to compare Fenton and Fenton-like processes for leachate treatment and the results reported are also controversial. Rivas *et al* reported that the Fenton and Fenton-like reactions had similar organics removal efficiencies, whereas Kim *et al* found that the Fenton reaction achieved a higher COD removal and a higher BOD_5_/COD than the Fenton-like reaction and the optimal pH = 3 for Fenton oxidation was below the optimal pH = 4.5 for Fenton-like reaction [18]. Finally, the BOD_5_/COD ratio increasing order of the applied AOPs in this work was photo-Fenton >Fenton >Fenton-like.

### Comparison of treatment processes

As shown in Table 2, in optimum conditions, the maximum and minimum removal percent of COD were in the photo-Fenton and Fenton-like processes, respectively. The maximum and minimum removal percent of TKN were in the Fenton and photo-Fenton processes, respectively. Also, the lowest and highest rate of phosphate removal was in the Fenton-like and photo-Fenton processes, respectively. In all processes, TKN removal in comparison with other parameters was low, and phosphate removal with over 95% was more than the other parameters.

**Figure 7:**
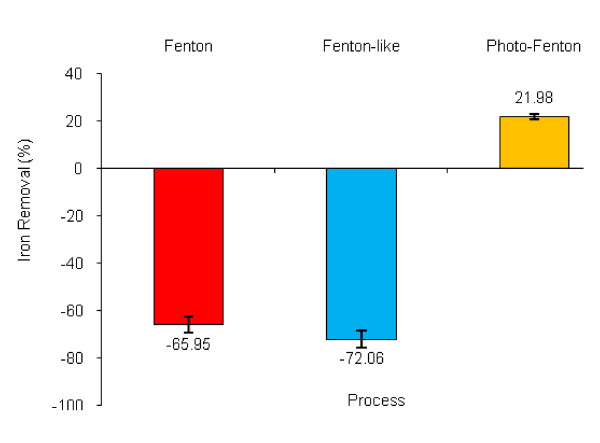
Variation of iron concentration in the treated leachate based on the studied processes

The amount of COD removal by Fenton, Fenton-like and photo-Fenton processes in various landfill leachate and molar ratio of H_2_O_2_ to Fe^2+^/Fe^3+^ in various studies are different, that is probably because of the differences in the characteristics of raw leachate and complexity of leachate compounds. In general, Cr removal was more than Cu removal after all processes. Information on the removal of other constituents (e.g., metal ions) from leachate by Fenton treatment is scarce. Although there is little information about the mechanism of the removal of metals, it seems that sludge sedimentation sweeps and removes the heavy metals from solution [18]. As Figure 7 shows, not only the iron concentration of leachate did not decrease after treatment by the dark processes (the Fenton and Fenton-like), but also its concentration increased. Iron concentration decreased only in the photo-Fenton process. Hence, adding iron as a catalyst in all processes leads to an increase in iron concentration in both the effluent and the produced sludge, that is one limitation for Fenton-based processes. In these processes, iron cycles between Fe^2+^ and Fe^3+^ plays the role of catalyst. In the photo-Fenton process, UV radiation can enhance the reduction of dissolved Fe^3+^ complexes to Fe^2+^, and generate additional OH radical via photolysis. As a result, the production of iron sludge reduced. This phenomenon has clearly discussed by other researchers [18]. 

The Fenton reaction can be suitable for the old leachate treatment compared with other AOP processes [7,18]. The Fenton process is technologically simple and there is no mass transfer limitation due to its homogeneous catalytic nature, also both iron and hydrogen peroxide are cheap and non-toxic [7]. The Fenton processes are applicable for the treatment of highly toxic leachate and noticeably exhibit faster kinetics as compared with the biological treatment processes. The Fenton treatment also achieves considerably higher efficiency of removal of organic compounds from leachate compared with other physical/chemical technologies including coagulation and activated carbon adsorption. In contrast to membrane filtration and other separation processes, the Fenton treatment affects chemical destruction rather than pollutant transfer from one phase to another. Importantly, the capital costs of the Fenton treatment can be expected to be very low in comparisoin with other advanced oxidation processes such as UV/H_2_O_2_[18]. An appropriate and economical method should be investigated and used for leachate treatment with Fenton and Fenton-like processes to remove the excess iron in effluent and iron sludge disposal. The Fenton-like process had the lowest efficiency compared to Fenton and photo-Fenton processes.

This investigation has led to the following results: Firstly, the efficiency order of the applied AOPs in this work regarding COD, TKN, phosphorus and heavy metals reduction was: photo-Fenton >Fenton >Fenton-like. Secondly, although the lower iron sludge is produced in the photo-Fenton process, in practice, maintenance and operation of UV lamp is difficult because of scale problems and high treatment costs due to high electricity consumption [20]. In addition, using UV lamp causes long-term health risks regardless of safety. With these conditions, it seems that photo-Fenton process cannot be used in large scale in Iran. Finally, considering the obtained removal percentages in the performed processes, it can be concluded that Fenton-like process had a lower efficiency than Fenton and photo-Fenton processes in the removal of leachate contaminats. But, the Fenton and photo-Fenton processes need low pH, so these processes consume large amounts of acid for adjustment of leachate pH before the process. pH adjustment significantly increased the effluent TDS and created operational and safety risks and caused corrosion. These are disadvantages of these processes [7,18], whereas the Fenton-like process is done at a higher pH. Therefore, appropriate process can be selected and used in full scale depending on the purpose of the leachate treatment, the efficiency of the required treatment, output levels of pollutants, characteristic of the leachate, landfill age, economic condition, investment and operational costs, operational complexity and circumstances of each country.

## Competing interests

We declare that the financial of this study has supported by the Research Deputy of Mazandaran University of Medical Sciences. We declare that we have no significant competing financial, professional or personal interests that might have influenced the performance or presentation of the work described in this manuscript.

## Authors’ contributions

The overall implementation of this study including design, experiments and data analysis, and manuscript preparation were the results of joint efforts by individuals who are listed as co-authors of this paper. All authors have made extensive contribution into the review and finalization of this manuscript. All authors read and approved the final manuscript.
